# Now is the time: operationalizing generative neurophenomenology through interpersonal methods

**DOI:** 10.1093/nc/niaf052

**Published:** 2025-12-27

**Authors:** Anne Monnier, Lena Adel, Guillaume Dumas

**Affiliations:** Precision Psychiatry and Social Physiology laboratory (A.17.004), Centre de recherche Azrieli du CHU Sainte-Justine, 3175 chemin de la Côte-Sainte-Catherine, Montréal (QC) H3T 1C5, Canada; Department of Psychiatry and Addictology, University of Montreal, Faculty of Medecine, Pavillon Roger Gaudry, Montreal, C.P. 6128, succursale Centre-ville Montréal (Québec) H3C 3J7, Canada; Precision Psychiatry and Social Physiology laboratory (A.17.004), Centre de recherche Azrieli du CHU Sainte-Justine, 3175 chemin de la Côte-Sainte-Catherine, Montréal (QC) H3T 1C5, Canada; Department of Psychiatry and Addictology, University of Montreal, Faculty of Medecine, Pavillon Roger Gaudry, Montreal, C.P. 6128, succursale Centre-ville Montréal (Québec) H3C 3J7, Canada; Integrated Program in Neuroscience, McGill University, McGill University 845 Sherbrooke Street West Montreal, QC H3A 0G4, Canada; Precision Psychiatry and Social Physiology laboratory (A.17.004), Centre de recherche Azrieli du CHU Sainte-Justine, 3175 chemin de la Côte-Sainte-Catherine, Montréal (QC) H3T 1C5, Canada; Department of Psychiatry and Addictology, University of Montreal, Faculty of Medecine, Pavillon Roger Gaudry, Montreal, C.P. 6128, succursale Centre-ville Montréal (Québec) H3C 3J7, Canada; Mila–Quebec AI Institute, University of Montreal, 6666 Saint-Urbain Street, Montreal, QC, H2S 3H1, Canada

**Keywords:** enaction, neurophenomenology, intersubjectivity, multi-brain neuroscience, multiscale modelling, dynamics of consciousness, computational psychiatry, complex systems

## Abstract

Lived experience is shaped by intersubjective, social, cultural, and historical dimensions. For the past 30 years, neurophenomenology has adopted an embodied perspective of the mind by integrating first-person experiential and third-person neurobehavioural perspectives. Neurophenomenology reveals mutual constraints between both, as they co-constitute a person’s lived experience. This article emphasizes the intersubjective and social facets of lived experience as core to generative neurophenomenology, envisioned in the 1990s by Francisco Varela, and argues that the scientific community is now ready to adopt this approach. For this endeavour, we clarify three meanings of ‘generative’ as it applies distinctly to generative phenomenology, generative passages, and generative models. Then, we propose to combine existing methods to update neurophenomenology program: first, by transitioning from individual to multiple people phenomenology methods that include intersubjectivity experience; second, by expanding traditional neuroscience to include measures of multimodal interpersonal synchrony; and third, by leveraging multiple computational tools to integrate different viewpoints, thereby enriching our understanding of lived experience. We also underscore the potential of diverse mathematical formalisms to capture aspects of human experience, all while underscoring that using computational approaches to model neurophenomenology does not entail endorsing computationalism as a grounding hypothesis of human experience. Finally, we illustrate the clinical relevance of this paradigm through two case studies in psychiatry—(1) with interactive dyads in autism and (2) with multiple members in family therapy sessions—demonstrating its translational potential.

## Introduction

‘This mind is that mind, cognition is generatively enactive in the co-determination of Me-Other’—*Francisco Varela* (Varela 1999b, p. 86).

Neurophenomenology is a methodological framework introduced by Varela that integrates first-person perspective (1PP) experiential data with third-person perspective (3PP) neuroscientific observations, treating consciousness as a domain that demands both phenomenological rigour and scientific tractability (Varela 1996). Even in its early formulation, Varela anticipated an extension beyond the individual level into the interpersonal domain which he termed ‘Generative Neurophenomenology’ (Varela 1999b). Drawing on Husserl’s notion of generativity, this extension was meant to emphasize the unfolding of meaning through interpersonal and cultural processes over time (Husserl 1960; Steinbock 1995).

At the time neurophenomenology was proposed as a paradigm, however, even thinking of cognition as anything but internal information processing was unconventional. As such, the empirical and conceptual tools required to study the relational dynamics that would turn neurophenomenology into generative neurophenomenology were not yet in place.

A crucial conceptual shift had nonetheless already been initiated nearly a decade earlier. Maturana and Varela’s (1992) theory of autopoiesis offered a biologically grounded account of cognition, describing living systems as self-producing, self-maintaining, and structurally coupled to their environments (see glossary, Autopoiesis). This view not only challenged the disembodied assumptions of the then-dominant computational paradigm (see glossary, Marr 1982) but put forward the notion that cognition (mind) and life are inseparable (Thompson 2010). By showing that cognition emerges through the same organizational principles that sustain biological life, autopoiesis laid a foundation for the integration of phenomenology, biology, and cognitive science. It opened the door for conceptual frameworks that have since made relational, situated, and embodied views of mind more acceptable within the cognitive sciences. Foundational work on embodied cognition (Varela et al. 1991), situated cognition (Smith and Semin 2007), intersubjectivity (Thompson 2001), 4E cognition—embodied, embedded, enactive, and extended (Newen et al. 2018)—and the skilled intentionality framework, which adds a fifth E for ecological to the 4Es (Rietveld et al. 2018; Troncoso et al. 2023), have not only expanded our understanding of mind as dynamically coupled with context, but also helped make such relational perspectives more accepted and tractable within cognitive science.

This theoretical groundwork provides an epistemic readiness for generative neurophenomenology which now converges with empirical advances. Recent developments in neuroscience, psychophysiology, behavioural science, and computational modelling enable researchers to study cognition as interactive and temporally unfolding. On the phenomenological side, methods have also matured, becoming accessible to non-expert participants and adaptable to dyadic and relational contexts, thus enabling systematic exploration of shared experience.

‘Now is the time’ marks this convergence of conceptual insight and technical feasibility. In this article, we show that generative neurophenomenology no longer remains a speculative ideal, but can serve as a viable paradigm for investigating consciousness as co-constituted, relational, and historically embedded. We do so by articulating a coherent account of generative neurophenomenology, outlining its conceptual roots (“What is generative neurophenomenology?”) as well as progressive methodological developments (“How can generative neurophenomenology be operationalized?”). This includes surveying phenomenological methods used to investigate intersubjective experience (“Operationalizing 1PP for generative neurophenomenology”). We then further provide an overview of methods to capture and analyse bio-behavioural markers of intersubjectivity (“Operationalizing 3PP for generative neurophenomenology”) as well as an overview of mathematical formalizations that afford linking first-person experience with third-person observational data in social contexts (“Computational approaches as the glue for generative neurophenomenology”). Finally, we explore implications for psychiatry (“Why does generative neurophenomenology matter for psychiatry?”) and provide descriptions of two of our own ongoing research projects as case examples illustrating how to apply a generative neurophenomenology paradigm in practice.

## What is generative neurophenomenology?

Generative neurophenomenology (GNPh) is a concept introduced by Francisco Varela (Varela 1999b, p. 86) in its nascent form. The term ‘generative’ used with neurophenomenology builds upon the tradition of phenomenology. To understand it, three foundational components are clarified in this section: the conceptual scope of generativity (“From phenomenology to generative phenomenology”), the methodological requirements of social approaches (“From neurophenomenology to generative neurophenomenology”), and a shared semantic grounding (see Box 1 and supplementary material). To address the last point, we provide a structured glossary in the supplementary material. The following section aims to clarify the semantic and operational distinctions between the various uses of the term ‘generative’ and to delineate the contours of what we refer to as generative neurophenomenology.

**Box 1: Clarifying the 3 meanings of the term ‘Generative’.**

Three current uses of the term ‘generative’ refer to conceptually distinct notions: generative phenomenology, generative passages, and generative models. Clarifying these distinctions is essential for ensuring conceptual precision moving forward in generative neurophenomenology (GNPh).

**Generative phenomenology (and GNPh)**

Rooted in Husserl’s later work (Husserl 1970; Steinbock 1995), generative phenomenology is the study of structures of consciousness as experienced from the first-person perspective (1PP), but going beyond static (internal-structural) and genetic (stream and transitional) dimensions of experience (Marín-Ávila 2025). Thus, it includes interpersonal (micro), historical (meso), and cultural (macro) levels of constitution, emphasizing the co-constitutive relationship between self and other. Generative phenomenology is inherently temporal, dynamic, and embedded in a social structure. It posits two intertwined timeframes: one immediate and situated in joint action (interpersonal coupling), and another extended across personal history, culture, and evolution. Varela (1999b) drew upon this tradition to propose a ‘second step’ in the neurophenomenological program: GNPh precisely refers to the definition of generative phenomenology.

**Generative passages (also called generative mutual constraints)**

This concept, introduced by Varela (1997) and further developed by Lutz (2002), refers to dynamic and reciprocal relations between domains (in particular first- and third-person perspectives) that are not merely logically consistent through mutual constraint, but are operationally generative, i.e. producing emergent properties that exceed the sum of their individual components through their circularity. In order to operationalize generative passages, the three following conditions must be met:

1. Formal precision, allowing for mathematical and scientific articulation;
2. Natural grounding, linking broad experiential phenomena to situated, embodied processes;
3. Experiential contact, where lived experience meets bodily and material reality.

Meeting these conditions affords a systematic description of generative passages and thereby a way to test neurophenomenology’s central hypothesis: how the scaffolding in time of lived experience and its physiological counterparts are linked through reciprocal constraints.

**Generative models (for neurophenomenology)**

In computational science, a generative model refers to a formal system capable of producing new data based on learned probabilistic patterns or rules. While all generative models are computational, not all computational models are generative. In the context of consciousness studies, it is crucial to first distinguish that a generative model is not equivalent to artificial consciousness (Van Rooij et al. 2023); and second, that one can employ generative models to explore phenomenological structures without endorsing computationalism, remaining within an enactive and embodied framework (Kiverstein and Rietveld 2018). Indeed, generative models should remain anchored in the principles of biology to stay consistent with the autopoietic roots of enactive theory. Operationally, these tools enable generating data as simulations: generate 1PP from prior 1PP structures (see glossary, Computational Phenomenology); co-generate 1PP and 3PP (Computational Neurophenomenology); generating 1PP and 3PP for multiple people by simulating interpersonal dynamics (Computational Generative Neurophenomenology).

### From phenomenology to generative phenomenology

‘You cannot be by yourself alone. You have to inter-be with every other thing.’—*Thich Nhat Hanh, The Heart of Understanding* (Hanh 1987).

In order to understand the concept of GNPh, we must begin by recalling where the term ‘generativity’ in this context was derived from generative phenomenology. Generative phenomenology (Husserl 1970, 1973) investigates the critical role of social interactions in generating human experience and consciousness (Marín-Ávila 2025). This approach integrates insights from intersubjectivity into phenomenology to understand how meaning is generated through interactions with others (Varela 1999b, p. 85). Within the enactive field, it later inspired the development of the concept of ‘participatory sense-making’ (De Jaegher and Di Paolo 2007), which foregrounds coordination dynamics and autonomy in interaction.

By generativity, Husserl referred to both the process of becoming—the temporal generation of experience within a subject’s life—and the transmission of meaning across generations, through historical and social movement. According to Steinbock (1995), four main dimensions structure this generativity: (1) Experience unfolds within a familiar ‘homeworld’ that is always situated in relation to an ‘alienworld’. Their co-relational dynamic shapes how meaning is socially and intersubjectively constituted. (2) Meaning arises from inherited traditions but is actively re-appropriated through participatory sense-making within shared cultural horizons. (3) The existential limits of an individual, its birth and its death, structure the historicity of experience, anchoring subjectivity within generational continuity and rupture. (4) Language is a vehicle of transmission and transformation, enabling shared meaning, culture, and intersubjectivity across time.

Taken together, these four dimensions suggest that concretely incorporating the ‘Other’ into the scientific study of consciousness requires attending to immediate social interactions (dimensions 1 and 2) as well as to the broader historical, existential, and cultural embeddedness of experience (dimensions 3 and 4). The relational and experiential field that emerges in interactions, the so called ‘interbeing space’ (see glossary), captures the co-created, dynamic space where mutual presence, shared intentionality, and participatory sense-making unfold. This interbeing space is a relational field that is not reducible to the internal states of either interacting person, but rather emerges between them, as a field of inter-relational becoming, where identities and meanings are fluidly negotiated in real time.

In an effort to connect Western and Eastern philosophy with cognitive science, Thompson (2001) provided the foundation for integrating the principle of generativity into the scientific study of consciousness almost 25 years ago. Since then, various research strands have begun tackling the multidimensional nature of intersubjective experience and investigating the co-emergence of self and other.

### From neurophenomenology to generative neurophenomenology

The next step in understanding GNPh is to revisit its foundation in neurophenomenology since it is entirely built on the foundational principles and methodological developments of neurophenomenology (Varela 1996; Lutz 2002). Neurophenomenology is grounded in the systematic investigation of subjective lived experience (first-person perspective, 1PP; see glossary) and its alignment with physiological dynamics (third-person perspective, 3PP; see glossary), offering the tools and principles upon which generative extensions can now build. With regard to 1PP, neurophenomenology explicitly addresses the challenge of suspending habitual judgements to access and articulate the fine structure of pre-reflective experience, through specific training, instructions, and methods designed to guide attention towards the how of experience. This includes practices based on phenomenological reduction, like micro-operations analysis, contemplative practices, or the use of explicitation interviews (Petitmengin 2006). To support this rigour, the field has developed an iterative research strategy known as ‘front and back loading’ (see glossary), a methodological approach deeply rooted in mixed-methods research traditions that combine inductive and deductive logic. It alternates between rich phenomenological explorations (helping to identify relevant experiential dimensions to be measured or modelled, front load) and more structured and time-sensitive self-reports (enabling refining the 3PP interpretations and identifying correlations, back load) (Gallagher 2003; Petitmengin and Lachaux 2013; Abdoun et al. 2021). The combination of these methods (‘thick-to-thin continuum’ in Berkovich-Ohana et al. 2020; and in Lutz et al. 2025, see glossary) enables more systematic correspondences between 1PP and 3PP than either of them alone, capturing both synchronic aspects (those present at a single moment in time) and diachronic aspects (how experience unfolds or changes over time) of lived experience (Gallagher and Sørensen 2006; Jachs et al. 2022 (preprint); Timmermann et al. 2019).

In terms of 3PP, various bio-behavioural markers and their dynamics have been studied alongside 1PP to identify core neural and bodily correlates of experience and describe fundamental physiological patterns of phenomena of consciousness. Notable examples include investigations of attentional phenomenology linked to mindfulness-related brain states (Lutz 2002; Lutz and Thompson 2003; Lutz et al. 2025), phenomenological preictal signs in epilepsy and their neural correlates (Le Van Quyen and Petitmengin 2002; Petitmengin et al. 2007; Petitmengin 2010), the loss of ego sense during psychedelic experiences with corresponding cerebral markers (Timmermann et al. 2019, 2023), and cardiophenomenology studies that combine heart rate variability with bodily emotion maps (Nummenmaa et al. 2014;  Colombetti 2014; Golland et al. 2015; Troncoso et al. 2023; Depraz and Desmidt 2019).

Such studies exemplify the productive pairing of 1PP and 3PP in neurophenomenology. A comprehensive list of >20 studies can be found in Berkovich-Ohana et al. (2020, Table 2).

While neurophenomenology has advanced the correlation of 1PP and 3PP, two key challenges remain. The first concerns the circularity hypothesis: it remains difficult to show that lived experience not only correlates with or emerges from neural and bodily dynamics, but also actively shapes physiological processes in return (Cleeremans and Tallon-Baudry 2022; Froese and Sykes 2023). Empirical evidence of this shaping is still scarce, with some pioneering work addressing e.g. the modulation of epileptic seizures by attentional experience (Le Van Quyen and Petitmengin 2002). Second, efforts to formalize ‘generative passages’ (see glossary) between 1PP and 3PP are still in their early stages on the individual level (Ramstead et al. 2022; Lutz et al. 2025).

In this paper, we describe the extension of the methodological foundations provided by the neurophenomenology research program towards the interpersonal realm, suggesting the same iterative and integrative rigour to the collection, analysis, and modelling of lived experiences, not only at the level of the individual, but within co-enacted social interactions involving multiple participants. In doing so, we aim to contribute a concrete step towards a GNPh framework, advancing our understanding of how shared meaning and lived experience dynamically unfold between multiple people.

This new framework aims to extend the working hypothesis of neurophenomenology to an angle that explicitly includes the Other, emphasizing the existence of social coupling (see Glossary, Autopoiesis).

‘Phenomenological accounts of the structure of experience and their counterparts in cognitive science relate to each other through reciprocal constraints’ (Varela 1996, p. 343).

becomes

‘**Generative** phenomenological accounts of the structure and dynamics of experience, and their counterparts in **social** cognitive neuroscience, relate to each other through **individual and social** reciprocal constraints**.**’

**Figure 1:**
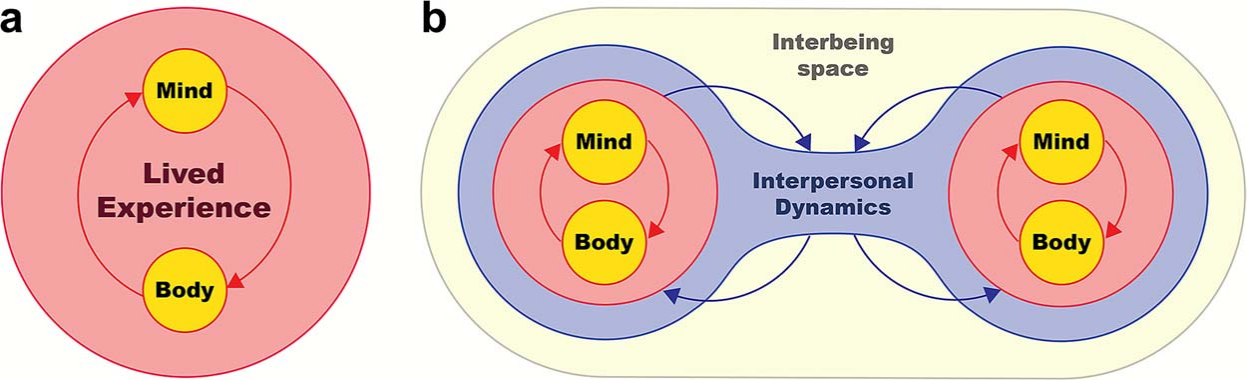
From neurophenomenology to generative neurophenomenology in a dyadic context. (a) At the individual level, lived experience emerges from the dynamic coupling between mind and body. First-person perspective (1PP) (e.g. self-reports) and third-person perspective (3PP) (e.g. physiological signals) are mutually constraining, forming a loop that supports neurophenomenological inquiry. (b) In dyadic interactions, each person’s mind–body system is coupled with the other’s, giving rise to a shared interbeing space. This space emerges from ongoing interpersonal dynamics. The outer arrows in b linking the circles which contain "Mind" and "Body" with the "Interpersonal Dynamics" structure indicate social coupling across individuals, leading to the emergence of the interbeing space (the outermost structure in b), which can in turn influence each participant’s internal processes. This dual loop—individual and interpersonal—forms the basis of generative neurophenomenology, a framework that views lived experience as co-shaped by both internal and relational dynamics. It aligns with the logic of autopoiesis, emphasizing self-organization within social coupling. While this model focuses on dyads, it can be extended to multi-person systems. This figure uses the ‘mind–body’ terminology to illustrate ontological rather than pragmatic aspects; limitations of the representation are notably the absence of temporal layers such as developmental, cultural, and historical context.

This reformulation shifts the focus to the interpersonal realm, actively including the study of social interactions that Varela intended but which was not yet empirically possible at the time. Neurophenomenology’s working hypothesis has, to date, been primarily interpreted as concerning the individual generative passages between phenomenological structures of experience and their physiological correlates (see Fig. 1). Pragmatically, however, adopting a working hypothesis grounded in generativity involves integrating generative phenomenology into neuroscience by: (1) extending the notion of generative passages from the individual to the interbeing level, and (2) explicitly addressing, within the research questions formulated, the circular relationship between individual and interpersonal generative passages. A GNPh approach aims to clarify how these two levels relate and mutually influence one another (see Fig. 1).

## How can generative neurophenomenology be operationalized?

**Figure 2:**
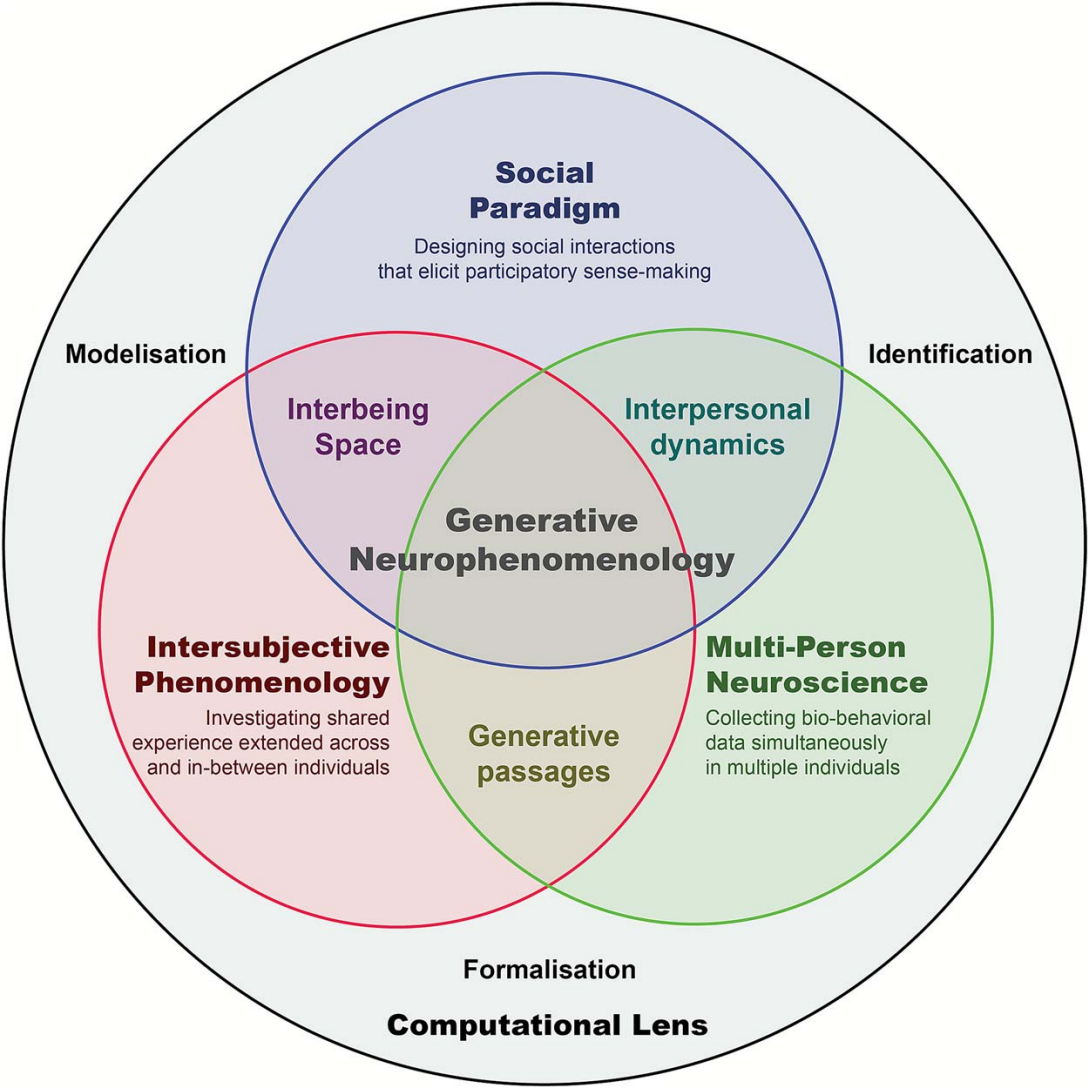
Building blocks for operationalizing generative neurophenomenology. This diagram illustrates four foundational components for studying shared experience within a generative neurophenomenological (GPNh) framework: The upper circle (social paradigm) represents the design of interaction contexts that elicit participatory sense-making, especially relevant for clinical and neurodivergent populations. The left circle (intersubjective phenomenology) represents the expansion of first-person methods to relational domains, capturing experience as co-constituted between individuals. The right circle (multi-person neuroscience) represents the integration of multi-brain and multi-body recordings to investigate dynamic interpersonal coordination. The outermost circle (computational lens) represents formal modelling and integration tools that bridge subjective reports with bio-behavioural data across scales. Together, these components structure the empirical and theoretical scaffolding of GNPh.

Observing and modelling an interbeing space and its generative passages is not trivial. It first requires empirically grounded, multi-person paradigms capable of enabling an authentic participatory sense-making, thereby illustrating the situated nature of agents within their environmental niche (Favela 2023). Each interactional paradigm comes with its unique methodological challenges and possibilities (Parada 2018). Second, it calls for rich and multiple phenomenological accounts of a shared intersubjective experience. Third, it necessitates multimodal data collection capturing bio-behavioural signals in parallel. A fourth, now critical building block for operationalizing GNPh is the integration of computational tools and formal models to extract meaningful patterns from the complexity and multidimensionality of such data. These foundational components are summarized in Fig. 2 and will be further developed in the remainder of this section.

### Operationalizing 1PP for generative neurophenomenology

#### Anchoring in best practices of phenomenology and contemplative and qualitative studies in social sciences

A core challenge of neurophenomenology, equally present in the GNPh framework that we propose, is developing systematic methods to make lived experience scientifically tractable, enabling bridging 1PP with 3PP (Varela 1997). This challenge was first addressed in contemplative science, building on the skills of meditators or trained participants to observe their experience. It was tackled by adopting rigorous methods such as microphenomenological interviews (MPIs) to identify fine-grained experiential invariants like subtle bodily sensations, attentional shifts, and pre-reflective awareness (Petitmengin et al. 2019; Berkovich-Ohana et al. 2020).

Methodological advances in 1PP collection have been structured along two main axes (Lutz et al. 2025). The first is the thick-to-thin continuum: thick methods, rooted in the phenomenological epoché (see glossary, FIrst-Person Perspective), yield rich, idiosyncratic descriptions (e.g. MPIs; Petitmengin 2006; Petitmengin et al. 2019), while thin methods rely on semi-structured or structured self-reports and experience sampling, making them easier to correlate with 3PP like physiological time series. Tools such as Likert scales or Temporal Experience Tracing further enable fine-grained tracking of experiential dynamics over time (Gernert et al. 2024; Jachs et al. 2022 (preprint)). The second axis concerns the unfolding of the temporal span of a specific experience, ranging from just-past moments to broader time windows extending to lifespan trajectories (Lutz et al. 2025).

Extending these methods from trained, highly skilled contemplative practitioners to the general public and untrained individuals, while also shifting the focus from an individual to an intersubjective experience, introduces both theoretical and methodological challenges. Yet, this shift has been enriched by importing the rigour and best practices of both phenomenological inquiry and qualitative social research (Valenzuela-Moguillansky et al. 2021; Timmermann et al. 2023), paving the way for capturing rich 1PP in more naturalistic social interactions (De Jaegher et al. 2017; Martínez-Pernía et al. 2023).

#### Enactive intersubjectivity in the intercorporeality

In the enactive framework, intersubjectivity is understood as a dynamic co-regulation of sense-making between agents, known as the participatory sense-making (De Jaegher and Di Paolo 2007; Fuchs and De Jaegher 2009; Tanaka 2017). It is conceived as a social coupling process where both agents influence and are influenced by each other, creating emergent patterns of interaction that are not reducible to individual intentions or behaviours. This interbeing space (Varela 1999b) develops autonomy and acquires its own dynamic structure through feedback and feed-forward loops of resonance and dissonance. Importantly, in this view, the dynamic interplay between attunement and alienation, synchrony and desynchrony, becomes constitutive of mutual understanding while preserving individual subjectivity.

In enactive intersubjectivity, this process of social coupling in the time and space of the interaction is described as ‘*mutual incorporation*’, in which the living bodies of both participants extend and form a common intercorporeality. It includes several components such as bodily resonance (Froese and Fuchs 2012), affective attunement (Seyfert 2012), joint attention, joint action, swaying postures, mutual gazes, coordinated gestures, facial expressions, or vocal tone (De Jaegher and Di Paolo 2007; Fuchs and De Jaegher 2009), which are observable as 3PP (further discussed in the section titled "Operationalizing 3PP for generative neurophenomenology"). The dynamics of these bodily resonance variations define the interbeing space which is an embodied relational field that is the primary site of interest for GNPh, as it both shapes and is shaped by each participant’s lived experience.

#### The interbeing space described as a multi-1PP domain

When collecting 1PP in a GNPh context, the aim is to gather phenomenological data that help us understand how each experience remains distinct while also being shaped by mutual incorporation. Phenomenological data are, by default, individually reported which raises a methodological limitation that needs to be acknowledged when studying intersubjective or shared experience: we cannot directly access a ‘plural’ or a joint subjective report. Lived experience, by definition, belongs to a single embodied subject. However, this does not rule out the investigation of intersubjective dynamics or generative passages between embodied agents. This raises fundamental questions about whether and how group or collective consciousness might emerge from these intersubjective processes (Kastel and Dumas, in preparation). These questions that remain largely unexplored in phenomenological research despite their potential significance for understanding shared intentionality and collective sense-making. A promising strategy involves collecting 1PP from all participants involved in the interaction, and also from an observer when possible, and analysing the commonalities and divergences of their descriptions. However, the interbeing space can sometimes be explicitly reported by a single participant (De Jaegher et al. 2017; Martínez-Pernía et al. 2023). In fact, this model of investigation has informed previous studies on intersubjectivity and offers a relevant and insightful framework for exploring the dimensions of multi-1PP in GNPh. Even if ideally, it is accessed through multi-perspectival first-person evidence.

#### Intersubjective dimensions and suggested coding matrix support

Since Thompson (2001), phenomenological investigations have highlighted key experiential modalities of intersubjectivity. A recent framework proposed by Troncoso et al. (2023) organizes 1PP on empathy into four operational axes: subjectivity, body, interaction, and context. This division offers a foundation for coding intersubjective themes within a GNPh framework. The first two axes are particularly relevant for informing individual generative passages, whereas the latter two support the exploration of interpersonal ones.

**Figure 3:**
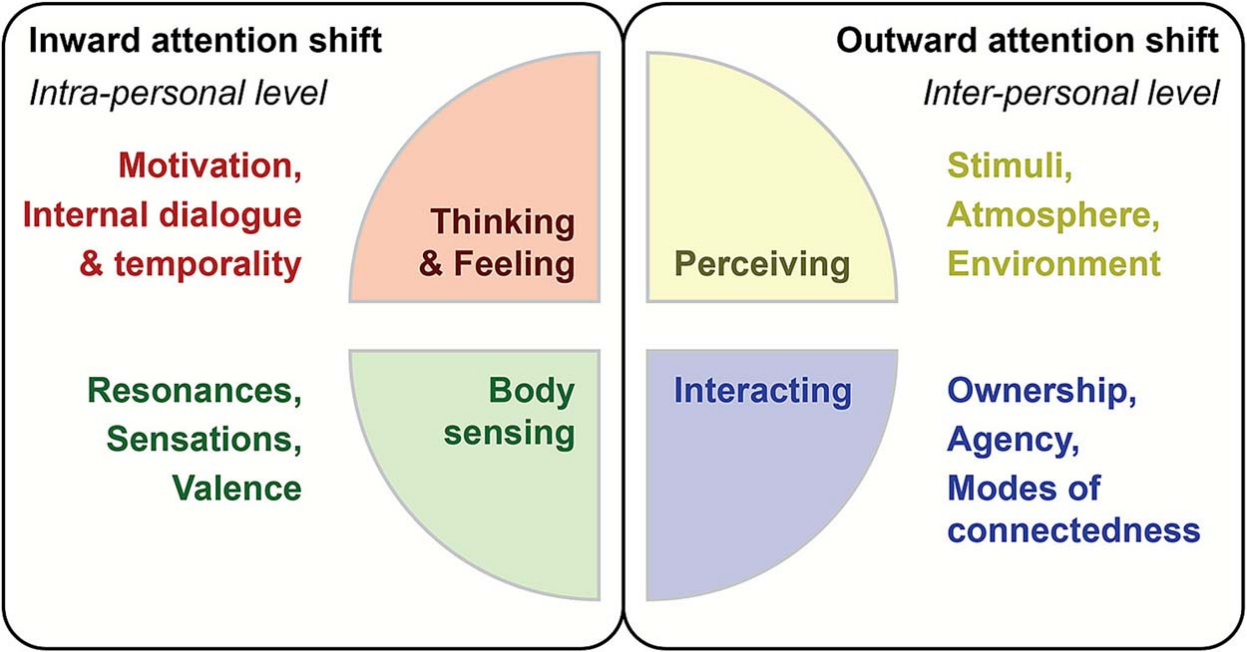
Intersubjective dimensions and suggested coding matrix. This figure, adapted from Troncoso et al. (2023), organizes first-person perspectives (1PP) along two attentional directions relevant to generative neurophenomenology. The left side represents an inward focus comprising two main axes—thinking and feeling (e.g. internal dialogue, motivation, temporality) and body sensing (e.g. bodily resonances, sensations, valence), based on the experiential levels from the PRISMA framework: thinking, feeling, and sensing (De Jaegher et al. 2017). These will be used to formalize individual generative passages by linking these codes to individual third-person perspective (3PP). The right side reflects an outward focus, giving insights into how the person is embedded in and influenced by the social interactions and the surrounding environment. This side is structured around perceiving (e.g. external stimuli, atmosphere, environmental perception) and interacting (e.g. shared engagement, relational dynamics such as ownership, agency, and modes of connectedness to the other). These dimensions assist in formalizing interpersonal generative passages and can inform phases of interpersonal synchrony and desynchrony within the interbeing space.

Extended work in the domain of pain empathy from Martínez-Pernía et al. (2023) and Zepeda et al. (2025) used further experimental phenomenological designs. This series of studies proposes a rich coding matrix identifying major relevant dimensions such as bodily resonance (including bodily sensations, affective quality, affective valence, kinesthetic aspects), motivation (including protective, altruistic, or absent motivation), internal dialogue (judgements and analysis), sense of ownership (self vs. other experience), and temporality (internal vs. external). We refined the four main codes originally proposed by Troncoso et al. (2023) categorizing them into two intrapersonal dimensions—Thinking and Feeling (subjectivity) and Body Sensing (body)—and two interpersonal dimensions—Interacting (interaction) and Perceiving (context). This reformulated division is our novel step towards distinguishing and organizing 1PP that inform or constrain intrapersonal generative passages, from those that more directly shape interpersonal generative passages—emerging within the interbeing space (see Fig. 3).

Actually, this reformulation presented in Fig. 3 does justice to the three experiential levels of intersubjectivity we draw from the seminal PRISMA framework for the systematic study of intersubjective experience (De Jaegher et al. 2017). Like a prism, PRISMA reflects multiple perspectives within an interaction—those of both interactors, as well as external observers. These perspectives are accessed either in real time or retrospectively through video annotation, using a structured method of analysis. Notably, interactive PRISMA workshops include guided observer inputs and investigate dynamic shifts between self- and other-perception. This framework directly builds on the theory of enactive intersubjectivity (De Jaegher and Di Paolo 2007; Di Paolo and De Jaegher 2019), by tracking transitions across three experiential levels of lived experience: thinking (cognitive), feeling (affective), and sensing (bodily).

To further support the structuring and inductive analysis of intersubjective first-person perspectives (1PP)—a crucial step before moving to a thin phenomenology—studies using MPIs of initial encounters in naturalistic settings have identified distinct modalities of agency and forms of intersubjectivity (Ollagnier-Beldame and Cazemajou 2019; Ollagnier-Beldame and Coupé 2019). These studies provide diagrammatic representations of shared experience using circles to represent the two agents and arrows to indicate the type and directionality of relational dynamics within dyadic interactions. A foundational and scalable tool in this visual tradition is the Inclusion of the Other in the Self scale (IOS, Aron et al. 1992), which uses overlapping circles to depict experiential states such as togetherness (a felt sense of co-presence, or ‘being one with another’) and connectedness (a felt sense of being linked or bonded to another person). Tools such as the IOS scale have demonstrated strong utility in large-scale ecological studies (Dikker et al. 2019, 2021) and may be effectively combined with tools like Temporal Experience Tracing (Gernert et al. 2024; Jachs et al. 2022 (preprint); Niedernhuber et al. 2023) to track fine-grained fluctuations in togetherness across time and activities. These methods allow precise tracking of connectedness dynamics and phenomenological shifts, particularly when applied during post-interaction video annotation (Noy et al. 2015). Examples of codes for the category ‘Interacting’—such as Ownership, Agency, and Modes of Connectedness—are also included in Fig. 3.

Though it still requires further refinement through thick phenomenological analysis and formalization, this methodological integration offers promising avenues for capturing both synchronic experiential units and the diachronic tipping points between intra- and inter-personal phases—thereby paving the way for a generative neurophenomenology of enactive intersubjectivity.

#### Limits and epistemic gain

In this article, we focus on methods for studying direct interactions between two or more individuals as they unfold over short time scales (seconds to minutes), with the aim of exploring how immediate social coupling shapes individual experience. This approach offers a practical entry point into the mechanisms of generative phenomenology. While intersubjectivity does not always require direct joint action (Zahavi 2023), we do not address longer-term forms of generativity, such as historicity and cultural transmission, which, although acknowledged as essential (Johansson 2011; Johansson and Emilson 2016), fall beyond the present scope.

Methods such as biographical narrative techniques appear promising for bridging psychosocial history with microphenomenological approaches (Depraz 2022; Sholokhova et al. 2022) including in the context of psychopathology (Henriksen et al. 2022). In addition, developmental psychology and, more recently, social and cultural psychiatry have begun to integrate individual and shared accounts of experience across broader temporal scales, further highlighting their relevance for psychiatry (Kirmayer et al. 2020; Gómez-Carrillo et al. 2023a; Kirmayer 2024) as further discussed in the section titled “Why does generative neurophenomenology matter for psychiatry?”

### Operationalizing 3PP for generative neurophenomenology

While the previous section focused on how 1PP can be extended to account for interpersonal aspects and for the interbeing space co-emerging as social interaction (see glossary, Intersubjective 1PP), this section surveys a selection of corresponding tools that afford us to study intersubjective processes from a 3PP. These interpersonal 3PP form the physiological scaffolding necessary for operationalizing a GNPh paradigm. The development of multi-body neuroscience methods, such as ‘hyperscanning’, dual physiological measurement, and behavioural coordination analysis, has enabled researchers to trace coupling dynamics across neural, physiological, and motor systems. Although still a niche within cognitive, affective, and social neuroscience, this work increasingly makes it possible to investigate intersubjectivity as a temporally unfolding, co-regulated process that builds on the notion that when people interact, their brains and bodies do not merely run in parallel, they enter into dynamic couplings. What distinguishes these approaches is not merely the inclusion of multiple participants, but their capacity to track how bodies and brains interact conjointly over time.

#### Multi-brain neuroscience

The emerging field of multi-brain neuroscience responds to the need for empirical methods that match the relational nature of intersubjective experience. Rather than treating individual brains as isolated units, this approach investigates how neural activity is shaped through dynamic, reciprocal engagement between interacting individuals. It builds on a growing body of work also referred to as two-body neuroscience, second-person neuroscience, or more recently relational neuroscience (Dumas 2011; Schilbach et al. 2013; De Felice et al. 2025). These frameworks emphasize that cognition and affect unfold not solely within individuals, but through the co-regulation of brains and bodies in real time. Within this perspective, hyperscanning, the simultaneous recording of multiple brains (Montague 2002), has emerged as a key technique. In its various forms (electroencephalography (EEG), functional near-infrared spectroscopy, functional magnetic resonance imaging, magnetoencephalography), hyperscanning captures brain activity across participants during live interaction, enabling analyses of inter-brain synchrony (Lindenberger et al. 2009; Dumas et al. 2010; Konvalinka et al. 2014). Evidence shows that such synchrony occurs across a range of social contexts and modalities, including face-to-face conversation, joint attention, and cooperative or competitive tasks, and is modulated by factors such as task structure (e.g. shared vs. complementary goals), emotional alignment (e.g. matched affective states), communicative intent (e.g. instructing vs. storytelling), and interpersonal rapport (e.g. familiarity or mutual liking) (Czeszumski et al. 2020; Hakim et al. 2023; Carollo and Esposito 2024). The underlying premise is that the transmission of information between brains follows similar principles to those that organize communication within a single brain (Varela et al. 2001). Accordingly, analytical tools originally designed for intra-brain dynamics, such as phase synchrony and functional connectivity, have been extended to model coupling across brains (Ayrolles et al. 2021; Dumas and Fairhurst 2021). This methodological bridge between individual- and multi-brain neuroscience has the potential to facilitate a generative turn in neurophenomenology by allowing direct reuse at the inter-brain level of the original measures designed for the intra-brain level.

#### Signal-processing methods for multi-brain neuroscience

Because intra- and inter-brain dynamics share common statistical properties, signal-processing techniques developed for single-brain analyses have been pivotal to the rise of multi-brain neuroscience. Phase-locking value (PLV) was among the first metrics to quantify phase alignment between neural signals, capturing the moment-to-moment coordination that Varela (1999a) linked to the ‘specious present’, the lived temporal thickness of experience (Lachaux et al. 1999; Varela et al. 2001). This breakthrough opened investigations into whether temporally coordinated neural assemblies support conscious episodes. Subsequent advances, such as the weighted phase-lag index (wPLI, Vinck et al. 2011), circular correlation (cCorr, Burgess 2013), and its recent refinements (Zimmermann et al. 2024), have increased the robustness and interpretability of interpersonal synchrony measures by reducing volume-conduction artefacts and spectral bias. Consequently, tools once reserved for intra-brain analysis now scaffold inquiries into embodied intersubjectivity (Dumas et al. 2010; Ayrolles et al. 2021; Zamm et al. 2024).

#### Multi-body neuroscience

Crucially, these advancements in signal processing at the multi-brain level are not limited to neural signals: they have also been used for investigating physiological rhythms (Konvalinka et al. 2023) and movement dynamics (Burger et al. 2014), demonstrating their cross-modal flexibility. This is of importance for GNPh, rooted in the enactivist view that cognition is constituted by sensorimotor loop activity. It follows that GNPh must also consider other embodied modalities of synchrony than the brain (McGann 2014; Newen et al. 2018; Troncoso et al. 2023) and make use of a ‘multi-body’ rather than solely multi-brain neuroscience (Dumas 2011). For example, movement synchrony, turn-taking in speech, eye movement, heart rate variability (HRV), and electrodermal activity (EDA) may also be central to how intersubjective processes unfold, offering additional windows into the dynamics of intersubjective 3PP beyond neural signals. These reciprocal dynamics generate the substrate from which shared understanding, mutual regulation, and meaning as described in the section titled “Operationalizing 1PP for generative neurophenomenology” can emerge in the interbeing space. Prior research shows that different behavioural and physiological modalities can tell us about various aspects of social interactions. For example, movement synchrony may provide insight into how coordinated physical activities like walking and dancing are linked to bonding, cooperation, and trust. It improves relationships and fosters prosocial behaviours (Wiltermuth and Heath 2009; Cirelli et al. 2014; Tunçgenç and Cohen 2016). Spontaneous movement coordination can reveal shared psychological states; linguistic synchrony seems indicative of mutual understanding and rapport (Gonzales et al. 2010; Fusaroli et al. 2012, 2014). Alignment in vocalization and speech patterns, such as turn-taking, fosters social cohesion and facilitates communication goals (Clark 1992; Pickering and Garrod 2004; Imel et al. 2014). The dynamic coordination of autonomic nervous system activity, or physiological synchrony, seems to be closely related to the emotional connections individuals form with each other. It includes the coupling of physiological signals like HRV and EDA. This synchrony is linked to better relationship outcomes and group performance in various settings, from parent–child interactions to therapeutic and team environments (Cacioppo et al. 2007; Feldman 2007, 2012; Palumbo et al. 2017; Mayo et al. 2021). GNPh calls for methods capable of measuring and modelling co-constitution across modalities.

#### Methods for cross-modal multi-body neuroscience

Multi-body neuroscience methods enable the exploration of intersubjectivity from a physiological processes perspective. To date, most studies have focused their attention on a single 3PP modality, leaving us with fragmented evidence. It is therefore unclear how these multiple markers dynamically interact, how they co-create an interbeing space, and how they relate to intersubjectivity for each participant’s experience. Overcoming this segmentation, e.g. by incorporating interpersonal movement and brain synchrony (Koul et al. 2023), necessitates a multiscale analysis (Delaherche et al. 2012, 2014; Kelso et al. 2013; Dumas et al. 2014). Multi-brain stimulation could also help causally manipulate the interpersonal 3PP dynamics, thereby facilitating the systematic assessment of the relationship of 3PP with 1PP (Moreau and Dumas 2021; Novembre and Iannetti 2021) which we should aim for in generative neurophenomenology. To model these dynamics as truly generative, we must treat their interactions not as parallel outputs but as recursive and asymmetric that mutually influence and transform each other. Tools such as transfer entropy (TE, Vicente et al. 2011), partial directed coherence (PDC, Baccalá and Sameshima 2001), and partial information decomposition (PID, Rosas et al. 2020) can capture directionality and multivariate coupling. These methods open a path to studying emergent dyadic phenomena where the whole may have causal properties not reducible to the sum of its parts (Luppi et al. 2023).

Furthermore, statistical inference must match the complexity of these systems. 3PP, especially when derived from multiple people across modalities, are often non-normal, interdependent, and high-dimensional. Non-parametric methods such as permutation testing and bootstrapping, along with cluster-based inference (Maris and Oostenveld 2007), provide robust options to handle this kind of data. Graph-theoretic extensions, including bipartite graphs for inter-brain data (Dumas et al. 2010), can further help in controlling multiple comparisons. Bayesian models (Diaconescu et al. 2017; Frässle et al. 2021) allow the incorporation of structured uncertainty and theoretical priors directly into the modelling process. They do so in three ways: by embedding theoretical assumptions formally, by modelling hierarchical and multiscale structures, and by supporting generative simulations of how intersubjective data might arise. Each of these features makes them especially well suited to the ambitions of generative neurophenomenology. In this way, Bayesian frameworks support theory-informed, multilevel modelling of experience as co-constituted across persons and time.

Machine learning, while often used in a bottom-up fashion, has added further capacity to interpret large-scale, multimodal datasets. Supervised approaches support outcome prediction, while unsupervised techniques uncover latent structure. Deep learning architectures are particularly suited for modelling nonlinear, high-dimensional data. Advances in natural language processing, including topic modelling, sentiment analysis, and representational similarity techniques, enable the systematic alignment of verbal content with physiological and neural data (Przyrembel and Singer 2018; Manuel et al. 2024). When used thoughtfully, these tools enable a more systematic analysis of how semantic, behavioural, and physiological patterns co-occur within interaction, compared to manual annotation or interpretive coding alone.

Together, these interpersonal multimodal 3PP methods do not merely enable us to analyse data, they shape the epistemic landscape for inquiry. They make it possible to study co-embodiment not as correlation or synchrony alone, but as a generative process unfolding across neural, bodily, and symbolic levels. In doing so, they fulfil the methodological prerequisites for a truly interactional science of consciousness.

### Computational approaches as the glue for generative neurophenomenology

In this section, we show how selected generative models, despite their formal diversity (Jebara 2004), can offer ‘epistemic gains’ (see glossary, Epistemic Gain) in GNPh by integrating 1PP and 3PP across interacting individuals. Given the current lack of consensus in consciousness science, a plurality of generative models is essential for formalizing both intra- and inter-personal data integration, each offering distinct insights into the generative links between experience and neural dynamics. Ultimately, using multiple formalisms enhances epistemic resilience and fosters conceptual flexibility, which is crucial for GNPh aiming to bridge lived experience and objective data in socially embedded contexts (Andrews 2020). We also aim to remind in that section that using computational tools does not entail embracing computationalism (see glossary). It is also important to keep in mind that ‘*The Math is not the territory*’ (Andrews 2020), meaning that the tools of analysis must not be conflated with the object of study itself. This caution became particularly salient with the recent development of active inference in neurophenomenology (Ramstead et al. 2022) and its combination of Bayesian formalism with explicit theoretical commitments (Andrews 2020).

Active inference models exhibit many properties that are attractive for computational neurophenomenology, particularly in formalizing hierarchical self-organization and coupling using Markov blankets. These Markov blankets can be nested (Ramstead et al. 2019; Ciaunica et al. 2023), and extended to model intersubjective dynamics, where inter-brain and physiological synchrony may serve as empirical proxies for message-passing across coupled Markov boundaries (Friston and Frith 2015). This computational formalism has already been applied to neurophenomenology (Ramstead et al. 2021) and is thus well positioned to be extended to generative neurophenomenology. However, we emphasize that this remains one possible formalization among others. Indeed, active inference reliance on top-down and discrete architectures also introduces limitations. First, while any computational model must define a structure to test hypotheses, active inference typically requires strong *a priori* specification of the state space and its transitions. This constraint limits its ability to account for emergent, unmodeled behaviours or novel system-level properties, which are central to socially extended and dynamically evolving cognition. In contrast, deep learning approaches, for instance, allow internal representations and transitions to emerge more flexibly through data exposure, supporting bottom-up formation of new representational layers in social contexts (Bolotta and Dumas 2022). Second, since active inference operates within a closed axiomatic and semantic system, it considerably limits falsifiability and interaction with other formalisms (Sheikhbahaee et al. 2023). For this reason, it is important to note that Bayesian statistics can totally be used to analyse and model first-person (1PP) and third-person (3PP) perspectives without strong theoretical commitment to the Free Energy Principle (FEP; Lee and Wagenmakers 2014; Ma et al. 2023). Active inference frames the behaviour of agents as if it results from Bayesian inference under a generative model, in accordance with the FEP. While this formalism has been employed in some theories of consciousness, such as predictive processing (Seth and Bayne 2022), the FEP itself is not inherently a theory of consciousness. Other frameworks also leverage generative Bayesian models to explore consciousness without assuming the FEP (Peters 2022).

Dynamical Systems Theory models can also present properties that expand our understanding of empirical data by showing continuity between qualitatively different states and mapping transitions between them (Kelso 1995). This bottom-up approach can uncover emergent properties, such as phase transitions, that are not present in the original model (Kelso et al. 2013). Although this approach does not directly address lived experience, models of nonlinearly coupled oscillators, like the Kuramoto (Kuramoto 1975) or Haken-Kelso-Bunz HKB models (Haken et al. 1985), are valuable for studying synchronization and coordination in complex systems. These models may offer concrete insights into how the temporal coordination of neural activities across brain regions (as well as between individuals) could underlie discrete moments of conscious experience, in line with Varela’s (1999a) concept of the ‘specious present’ as a dynamic, temporally extended but non-continuous window of awareness. Their scale-free property helps bridge different scales of description without reducing one to the other, describing dynamic modes homologous between observation angles and highlighting transitions observable at both brain and body scales (Kelso et al. 2013), and has the potential to formalize phenomenological dynamics and their interpersonal modulations. In GNPh, these models can incorporate social dimensions, extending beyond individual brain models (Dumas et al. 2012). Finally, these models can help explore the efficacy of consciousness and potentially address the impact of social interaction on intra-individual experience (Sánchez-Cañizares 2024).

Information theory offers a cross-domain formalism to address subjective richness, ineffability, and their relationship with interpersonal communication. For instance, the ineffability of subjective experience has been tentatively modelled as an information loss (Ji et al. 2023). The richness of a conscious experience then corresponds to the amount of information in that state, and ineffability can be seen as the amount of information lost at different stages of cognitive processing. Attractor dynamics in working memory, which stabilize neural activity patterns, would thus result in impoverished recollections of original experiences, contributing to ineffability. Additionally, the discrete symbolic nature of language fails to capture the high-dimensional structure of experiences, further enhancing the ineffability. By framing these concepts in an information theoretic perspective, this model highlights that much of the subjective experience remains elusive not because it is beyond scientific understanding but because of inherent information processing constraints within the brain. Moreover, this idea can be extended to the intersubjective dimension of experience (Ji et al. 2023). For instance, intersubjective ineffability may arise when individuals attempt to communicate their rich, subjective experiences to others, leading to further information loss due to differences in cognitive frameworks and linguistic limitations or synchrony potential. Thus, the model not only addresses the richness and ineffability of individual experiences but can also shed light on the challenges of sharing these experiences across different minds.

Overall, the key challenge for generative neurophenomenology is to evolve from descriptive mappings to explanatory formalisms that simulate and predict multi-mind–body systems. This may ultimately require the development of new mathematical formalisms, such as those invented to explain quantum phenomena, which provide epistemic gains (see glossary), particularly regarding the non-commutativity of conscious states (Busemeyer and Wang 2015; Tsuchiya et al. 2024) and the incorporation of historicity (Iriki and Taoka 2012; Iriki and Tanaka 2024). Finally, better integrating biophysical models seems essential for achieving comprehensive multiscale biological anchoring (Volzhenin et al. 2022). Modelling at the biophysical level does in fact allow to integrate neuropharmacology and how micro-scale modulations can change the macro-scale dynamics. It can therefore help to understand the links between changes simultaneously observed in brain dynamics and subjective experience, e.g. in psychedelic research (Bressloff et al. 2002; Petitot 2003). Ultimately, this may have important implications for psychiatry, helping to connect biological, experiential, and social dimensions of mental health without necessarily reducing one to the others.

## Why does generative neurophenomenology matter for psychiatry?

GNPh offers psychiatry a way beyond its long-standing epistemic divide: the gap between biological reductionism and the lived, relational nature of mental suffering. By integrating neurobiological processes, embodied experience, and social embeddedness, it provides a powerful framework for modelling psychiatric conditions as disruptions in interpersonal sense-making. Crucially, this shift aligns with the cultural-ecosocial systems approach for psychiatry (Gómez-Carrillo et al. 2023a) which views mental disorders not simply as brain-based dysfunctions but as breakdowns within ecologies of meaning-dynamic systems of relationships, institutions, and cultural practices that shape identity, agency, and vulnerability (Kirmayer 2024). Mental health emerges not from biology alone, but from the capacity to co-create meaning within a shared world. For practitioners, this demands a holistic and pragmatic orientation, grounded in enactive thinking that connects factors across scales—from neurotransmitter imbalances and trauma to existential distress and social exclusion, through the organizing principle of sense-making (de Haan 2020).

Systems neuroscience supports this multilevel view, showing how brain function arises from interactions across scales, from local neuronal dynamics to global networks (Le Van Quyen 2011; van den Heuvel et al. 2019). Multi-brain neuroscience extends this logic between brains, revealing that social engagement involves neural synchrony across individuals, with some inter-brain dynamics often disrupted in psychiatric conditions (Bilek et al. 2017; Moreau et al. 2024). Linking second-person neuroscience and social psychiatry reconceptualizes psychiatric disorders as disruptions in participatory social interaction rather than individual deficits or passive social perception. It frames conditions such as depression, autism, and schizophrenia as impairments in the dynamic processes of co-regulation, mutual engagement, and shared intentionality that underlie meaningful human connection (Schilbach 2016). Research makes these relational impairments measurable, providing new tools to track and potentially restore disrupted sense-making (Dumas 2022; Sened et al. 2022). Importantly, these ideas are not new. The Cambridge model of psychopathology long ago proposed that symptoms that emerge as biological signals are filtered through social interaction and cultural meaning (Berrios 1984). This view is now central to cultural psychiatry, which shows e.g. how voice-hearing varies across cultures—not just in content, but in distress and interpretation (Luhrmann et al. 2015; Kirmayer et al. 2017; Dumas et al. 2020; Veissière et al. 2020; Gómez-Carrillo et al. 2023b).

In sum, GNPh matters for psychiatry because it gives scientific insights to the field of psychiatry to reconceive mental disorders partly as failures in relational world-making. It offers a rigorous, integrative account of mental health as a multiscale, culturally mediated process of shared meaning.

### Case examples

To illustrate how GNPh offers a scientific toolkit enabling guiding empirical research in the field of social psychiatry, we present two case examples that demonstrate its applicability and relevance for psychiatry research.

In the context of autism, an inter-personalized psychiatry approach suggests that computational simulations of social interactions can provide complementary insights than analysing single-brain data alone (Bolis et al. 2022). These simulations can model differences in social interaction and meaning-making in autistic individuals, supporting new perspectives on neurodiversity. Rather than framing autism as an individual deficit, this approach highlights relational atypicalities as a more accurate and constructive lens (De Jaegher 2013; Mottron 2016; Green 2023).

One promising line of research developed in our laboratory at CHU Sainte-Justine in Montreal (2024) explores the experiential and physiological scaffolding of togetherness in neurotypical and autistic paediatric populations. The study investigates how togetherness is lived and co-enacted in real-time interactions between mother and child. Dyads engage in cooperative social activities adapted to engage autistic children (e.g. through the use of pictograms). Afterwards, they are invited to reflect on their lived experience of togetherness through the use of thin and thick phenomenological tools: (1) the Inclusion of Other in the Self (IOS) scale, tracking activity-by-activity variations in perceived closeness; (2) a body mapping activity, where each participant draws the most salient moment of togetherness on a two-body outline, capturing both inward and outward attention fields; and (3) microphenomenological interviews designed to access the fine-grained texture of the shared experience, offered to both mothers and—when possible—the children. These tools foster a reflexive stance, enabling participants to articulate subtle shifts in their relational experience. In this context, mothers often play a crucial role: their relational sensitivity and familiarity with their child’s unique modes of connection position them as situated experts. While they are not formally trained observers, their everyday attunement provides essential access to intersubjective 1PP. On the 3PP side, this study investigates how multimodal synchrony (across neural, physiological, and behavioural signals) relates to self-reported togetherness and social trait measures. A temporal modelling approach will further assess whether subjectively reported togetherness aligns with transient bursts of synchrony. Overall, this multi-person, multimodal approach offers a concrete example of how GNPh can be operationalized, not to measure individual deficit, but to illuminate the diverse relational dynamics that shape human experience in psychiatric conditions (see Table 1, example 1).

**Table 1:** Applying generative neurophenomenology to two clinical case studies.

	**Togetherness and interpersonal dynamics in mother–child dyads in autism**	**Rupture and repair strategies in the therapeutic alliance in family therapy**
**Exploratory Questions**	Do peaks in physiological synchrony correlate with self-reported or behaviorally marked moments of togetherness in mother–child dyads including autistic and neurotypical children?	Can rupture/repair transitions in therapy (as determined by trained therapists) be reliably detected through multimodal synchrony markers?
**Social Paradigm** *Experimental Setup*	Dyadic lab activities: mirror game, pictogram-based verbal sharing, resting states; Multi brain–body recordings: EEG, electrocardiograms, cameras	Real-life systemic family therapy sessions with up to five family members; Multimodal body recordings: wrist-worn sensors for physiological signals and cameras
**Multi-person Neuroscience** *Third-person data*	Neural and physiological synchrony metrics: PLV, HRV; Behavioural coordination: mimicry scores, mutual gaze, turn taking, temporal fluctuations and inter-modality predictive links	Physiological synchrony: EDA, HRV, body temperature; Behavioural coordination: movement, speech, language
**Intersubjective Phenomenology** *First-person data*	Thin: IOS scale ratings after each activity and brief identification of salient moments of togetherness; Thick: body mapping and microphenomenology interviews oriented toward interactional dimensions	Thin: short debriefs with therapists and families after each session; Thick: video-assisted coding by two trained therapists *post hoc*, independently and then discussed in focus groups
**Computational Lens** *Identification, modelisation, formalisation*	Data-driven modeling of synchrony dynamics; Information-theoretic measures of higher-order statistics (*e.g.* causal decoupling); Dynamical systems modeling of togetherness	LLM-assisted analysis of linguistic alignment and emotional content from session transcripts; Dynamical systems modeling of rupture/repair; Network modeling of group dynamics
**Possible Outcomes**	Support for non-deficit-based frameworks of autism by identifying alternative forms of relational attunement	Identification of embodied markers of therapeutic turning points with implications for supervision and therapist training

Beyond insights on the mechanisms underlying mental health conditions, considering interpersonal and social dimensions can also benefit conceptualizing therapeutic mechanisms like therapeutic alliance, which refers to the collaborative relationship between therapist and patient (Bordin 1979). Therapeutic or clinical alliance is one of the most robust predictors of treatment outcomes in mental health interventions (Flückiger et al. 2018) and can be modulated by cultural factors (Vasquez 2007). Incorporating interpersonal measures could prove particularly useful for types of therapy which involve understanding the patient’s social context. Systemic family therapy, for instance, focuses on the dynamics within the family system and how they contribute to the patient’s mental health (Gurman 1983; Carr 2005). In such conjoint treatments, the therapeutic alliance is not limited to a single dyadic relationship but forms a network of interrelated alliances between the therapist and each family member, and among the family members themselves. This complexity is further shaped by factors such as trust, conflict, loyalty, power dynamics, and secrets within the family system (Sprenkle and Blow 2004; Friedlander et al. 2006; Pinsof et al. 2008). Therapists must therefore attend to each relational strand, fostering balanced alliances while preventing split allegiances and supporting the development of a shared therapeutic focus. Another case in which we have begun applying GNPh in our research in Montreal as a guiding framework is a study on systemic family therapy for adolescents with complex mental health presentations. We investigate how therapeutic alliance as a dynamic, co-regulated phenomenon emerges and fluctuates through the embodied and semantic interplay between family members and their therapist. To operationalize this, we collect physiological 3PP (cardiac and electrodermal activity) from up to five family members and the family therapist using mobile sensors throughout the session. In parallel, we derive proxy-1PP by having the treating therapist as well as one other trained systemic family therapist annotate transcripts of the sessions with respect to therapeutic alliance-building, -rupture, and -repair, all of which are central to therapeutic change but difficult to capture through self-report because they are highly time locked. We complement this with sentiment analysis and linguistic alignment analysis using LLMs to explore how affective tone and verbal coordination shape and reflect moments of alliance. Rather than isolating individual experiences, we treat these annotations of alliance as situated traces of participatory sense-making. This allows us to examine how moments of interpersonal synchrony (measured in autonomic rhythms, turn-taking, and narrative structure) coincide with qualitative shifts in the therapeutic relationship. As GNPh demands, we approach these multi-person interactions not as additive, but as emergent interbeing spaces in which mutual incorporation gives rise to new affective and epistemic configurations. Our aim is to develop tractable models that respect the irreducibility of each participant’s perspective while foregrounding the co-constitution of experience in relational therapeutic work.

Incorporating neurophenomenological approaches into mental health research can lead to specific synergies between epistemic and therapeutic processes; combined with computational formalism, it can allow for a disciplined circulation between 1PP and 3PP, providing a framework to test not only mechanistic hypotheses but also therapeutic predictions (Lutz et al. 2025). The hope is that these updates on neurophenomenology will provide useful conceptual and methodological tools to integrate, investigate, and mutually constrain first- and third-person dimensions in mental health (Kyzar and Denfield 2023), and ultimately be relevant for addressing the current ‘crisis of mechanisms’ in psychiatry.

### Limitations, reflections, and considerations

We have outlined two case examples from the field of psychiatry to illustrate how GNPh can inform empirical research. As scientists working within a clinical and experimental tradition, we have focused on translating the paradigm into feasible research designs that address real-world concerns in the field of mental health. While these examples demonstrate concrete applicability, they necessarily reflect a partial view of what GNPh could encompass. The broader conceptual and methodological landscape extends far beyond what we could address here.

Our first limitation concerns temporality. The cases we discuss focus on real-time or short-term co-regulatory dynamics. Yet, Husserl’s account of generativity foregrounds historicity, not only in terms of biographical time, but across social and cultural generations (Steinbock 1995). Fully engaging this dimension will require longitudinal, developmental, and cross-cultural extensions of the current paradigm.

Second, but linked to the first point, although our cases address social interaction as a key site of dysfunction and therapeutic potential, echoing proposals that psychiatric disorders may be understood as disruptions of social coupling (Schilbach et al. 2013; Schilbach 2016), this framing may be too narrow. Social interaction unfolds within broader ecological and material niches. Individuals do not only attune to other people but to entire fields of affordances (Rietveld et al. 2013). Dysfunction may involve failures of world-integration as much as interpersonal attunement. A more ecological view of coupling will be needed to adequately model these dynamics.

Finally, we recognize that the very use of GNPh changes the research context. Our paradigm does not merely observe but participates in shaping meaning-making processes. This reflexive quality, where researcher and object of study are entangled, must be acknowledged and ethically addressed. The tools and models we use help bring forth the very dynamics we aim to describe (Barad 2007). This is especially relevant in psychiatry, where classificatory frameworks can feed back into lived experience, shaping how individuals understand themselves and are understood by others (Hacking 1995). Reflexivity is not a limitation to be corrected but a condition of inquiry that must be critically embraced (Frank et al. 2024).

## Conclusion

‘*Consciousness is a public affair*’, Francisco Varela (1999b).

In this article, we articulated how generative neurophenomenology can serve as both a conceptual and methodological framework. We began by situating the term within its historical and theoretical context, clarifying how it builds on and extends neurophenomenology through the integration of intersubjectivity and temporality. To address ambiguities surrounding the term ‘generative’, we clarified its use across three distinct yet interconnected meanings in this context: generative neurophenomenology, generative passage, and generative model. Subsequently, we surveyed how established methods in subjective reporting and neurobehavioural measurement have been adapted to investigate shared experience. We further highlighted the role of computational tools in linking data across first-, second-, and third-person perspectives, offering a cohesive structure for multilevel analysis.

While we consolidated and reframed existing work in the first two sections, in the final section we introduced concrete case studies, in paediatric autism and in family therapies, from our own work to illustrate two of many ways how generative neurophenomenology can be operationalized in empirical research. These examples demonstrated the paradigm’s utility for studying relational dynamics that are central yet difficult to capture with conventional approaches.

Our central claim is simple: *now is the tim*e. The conceptual foundations are in place; the tools needed and accompanying methods exist. What is needed is a shared vocabulary, practical orientation, and commitment to studying experience as something inherently relational and with this article we hope to contribute to meeting these needs.

In making the case that *now is the time* for operationalizing generative neurophenomenology, we have outlined the conceptual and methodological landscape that enables the study of consciousness as inherently relational, embodied, and temporally unfolding. Lastly, it is important to mention that this endeavour is not merely technical, it is also epistemological and communal. As Varela (1999b, p. 82) put it, ‘Examined experience and scientific analysis can have an explicit, non-dual relationship, a mutual determination, a circulation that avoids the extremes of both neuro-reductionism and some ineffability of consciousness.’ The slogan ‘consciousness is a public affair’ therefore refers not only to the intersubjective nature of consciousness itself, but also to the communities of inquiry that co-construct its scientific exploration. We believe that with generative neurophenomenology, we take a step towards realizing that vision: grounding subjective experience in rigorous empirical frameworks, while remaining responsive to the co-created, relational nature of meaning itself.

## Supplementary Material

Supplementary_materials_niaf052

## Data Availability

The data supporting the findings of this study are not publicly available, as this article is based on a conceptual and methodological analysis and does not include original empirical data.
