# Supplementary material for: Now is the time: operationalizing generative neurophenomenology through interpersonal methods
Source: Neurosci Conscious. 2025 Dec 27;2025(1):niaf052. doi: 10.1093/nc/niaf052 (PMC12743303; doi:10.1093/nc/niaf052)
Supplement: Supplementary_materials_niaf052 [file supplementary_materials_niaf052.docx]

**Supplementary material - Glossary of relevant terms**

The field of neurophenomenology draws on multiple disciplinary traditions, including philosophy, neuroscience, cognitive science, computational and mathematical fields, psychology, and psychiatry. As such, it involves a range of technical, conceptual, and sometimes ambiguous semantics whose meanings can vary depending on context.

To support clarity and transparency, this glossary aims to define relevant terms used in the article, outline their origins and disciplinary backgrounds, and summarize how they are applied within the scope of this article.

The entries below present the core themes and concepts essential to understanding the generative neurophenomenology framework. Within each definition, key notions supporting these main themes are highlighted in **bold**. While some terms are central to the article's argument, others appear more occasionally. All terms are listed in alphabetical order.

**Autopoiesis** (Maturana and Varela, 1992): Refers to the self-producing and self-maintaining nature of living systems. It implies both material autonomy (organisms maintain their own structure through internal processes) and informational autonomy (they generate their own meaning rather than passively receiving inputs).
While Maturana and Varela originally introduced structural coupling as a unified concept, later elaborations have distinguished orders of coupling to reflect increasing levels of systemic complexity:

- **First-order structural coupling** refers to the basic biological self-maintenance of an organism through self-organizing processes that regulate its internal milieu while remaining responsive to environmental perturbations.
- **Second-order structural coupling** emerges in multicellular organisms where specialized cells coordinate to form integrated systems like the nervous and immune systems. This enables more sophisticated forms of environmental coordination and internal integration.
- **Third-order structural coupling** (or ”social coupling”; Dumas, 2011) involves social interactions between autonomous agents that produce shared meanings, cultural practices, and collective behaviors. At this level, cognition and lived experience are co-constructed within social and cultural contexts.

**Computational Phenomenology** (Ramstead et al., 2022): A research approach that explores how subjective experience can be modeled using computational tools, like digital collection and signal or language analysis tools (e.g., ICA, transformers, LLMs), and mathematical formalisms aiming to bridge first-person perspective with brain dynamics (dynamical bridge). These computational tools include simulations, modeling frameworks (computational models), or algorithms that implement these formalisms to analyze or predict behavior and neural data.

**Computationalism** (Marr, 1982; Fodor, 1975): A theoretical view that treats the mind as a system performing computations on representations. It explains cognition as information processing, where mental operations are carried out by algorithms acting on structured internal representations.

**Epistemic Gain** (Lutz et al., 2024): Epistemic gain refers to the increase in understanding, insight, or explanatory power that results from a particular method, framework, or integration of perspectives. It denotes a situation in which new knowledge is not simply added, but where a richer, more coherent, or more comprehensive understanding of a phenomenon becomes possible. In neurophenomenology, epistemic gain emerges, for example, when subjective experience and objective data mutually inform one another in a way that neither domain could achieve alone.

**First-Person Perspective (1PP)** (Varela, 1996): In the neurophenomenological framework, first-person data refers to subjective accounts of lived experience, reported by a conscious subject who perceives, feels, and can articulate these experiences. These are first-person events, meaning they occur for someone - a subject or self. First-person perspective can be collected on a **“thick to thin continuum”** (Berkovich-Ohana et al., 2020):

- *Thick descriptions* involve rich, detailed accounts obtained via open-ended interviews or immersive fieldwork. Such data are best elicited through rigorous phenomenological methods, such as microphenomenology (formerly called explicitation interviews), which aim to access pre-reflective experience. These methods involve applying **phenomenological reduction** (Epoché, Conversion, Eidetic Variation) to suspend natural attitudes and allow access to the structural features of experience. This level of description, ordinarily obscured by the natural attitude, is immanent to lived experience. Phenomenological analysis renders explicit the structural dynamics of experience for the identification of **variants** (context-sensitive features) **and invariants** (stable features) of experience (in Husserlian terms), including both **synchronic features** (those present at a single moment in time) and **diachronic trajectories** (how experience unfolds or changes over time) of lived experience.
- *Thin descriptions* include brief ratings or structured reports along predefined dimensions arising from Thick descriptions. When scientifically analyzed using systematic methods, this data allows for a temporal bridge with 3P data.

**Front and back loading** (Gallagher, 2003): Front and back loading in neurophenomenology refers to an iterative research design that integrates first-person (1PP) and third-person (3PP) perspectives by alternating between two phases:

- Front-loading: Phenomenological insights (from interviews, experiential methods, etc.) are used before designing the experiment. This helps shape the experimental setup by identifying relevant experiential dimensions to be measured or modeled (e.g., attentional fluctuations, emotional tones, sense of self, etc.).
- Back-loading: After collecting 3P data (neurophysiological measures, behavioral markers, etc.), researchers return to the first-person level, often with new or repeated interviews or questionnaires, to refine the interpretation of the data and identify correlations.

This approach, emphasized by Gallagher (2003), Petitmengin (2010), and Lutz et al. (2015), helps overcome the disconnection between subjective experience and objective measures. It supports the “mutual constraints” principle: experience informs experimental design, and results refine experiential understanding. In generative neurophenomenology, as in neurophenomenology, the design of such a paradigm is not linear and can be done as sequential steps, in a front and back load design.

**Generative Models:** A computational specification of the mechanisms by which a system gives rise to its observable data. If the model is rich enough, the simulation reproduces the structure and dynamics of the real phenomenon. In our context, such models formalize the mapping between physiological processes and first-person experience, allowing hypotheses to be tested across these levels. When analysed within a Bayesian framework, the same formulation also yields a principled measure of model evidence, i.e. how strongly the observed data support a given model.

**Generative Neurophenomenology (GNPh)** (Varela, 1999b): A research program implying a broader neurophenomenology approach that explicitly incorporates social, interpersonal, historical or neurodevelopmental and cultural dimensions into the study of consciousness. It goes one step further than standard neurophenomenology by incorporating generative passages and data collection on two or more people forming a dyad or a social group of study. In this sense (and also because it builds on mathematical models and computational tools), it extends beyond the 5E cognition approach (embodied, embedded, enacted, emotional, and extended perspectives of empathy), including the neuro-physio-socio-phenomenology methodology.

**Generative Passages** (Varela, 1997): A formal construct describing the epistemically productive exchange between phenomenology, formal modeling, and biology*.* The concept of generative passages goes beyond merely establishing logical consistency between domains; it proposes that mutual constraints must be operationally generative. This means that there must be an active, reciprocal exchange that shapes the entire phenomenal experience. Such passages allow both reductionist analyses and phenomenological descriptions to be grounded in biological emergence, provided that three key dimensions are in place:

1. Formal precision for mathematical descriptions
2. A natural process linking broad phenomena to local sources
3. A pragmatic locus where lived experience meets bodily and material reality

Together, these enable mutual constraints to act as **generative passages**, bridging phenomenology and biology. This underlies Varela’s **working hypothesis** of neurophenomenology: *“Phenomenological accounts of the structure of experience and their counterparts in cognitive science relate to each other through reciprocal constraints.”* The terms **mutual constraints** or **reciprocal causation** are sometimes used in this context and point to the **circular nature** of both accounts, an unresolved middle ground encompassing their **bridges but their differentiations**.

**Generative Phenomenology** (Husserl, 1966, 73; Steinbock, 1995): A branch of phenomenology that examines how consciousness is shaped not only individually but also socially, historically, and culturally. It builds on **static phenomenology** (which examines the structural features of intentional experience at a given moment) and **genetic phenomenology** (which examines the genesis of meanings of things within one’s own stream of experience), and adds the **generativity** dimension.

**Generativity** (Husserl, 1966, 73; Steinbock, 1995): A concept introduced by Husserl which refers both to a process of becoming ("generation") and to a process that occurs *over generations*—specifically, the process of historical and social movement. According to Steinbock (1995), generative phenomenology is structured around four core ideas:

1. Phenomenological co-relativity of homeworld/alienworld (social)
2. The problem of constitution: sense stemming from tradition and sense-appropriation (participatory sense-making)
3. Birth and death as world-constitutive features (historicity)
4. Language and communication (transmission, culture)

As mentioned by Varela (1999b), generative phenomenology can be given a notorious philosophical foundation; they are the hidden iceberg of Husserl’s later works, slowly emerging thanks to the publication of his massive studies on constitution and intersubjectivity.

**Interbeing Space** (Thích Nhất Hạnh, 1980; Thompson et al., 2001): A relational space that emerges dynamically between individuals through embodied social interaction. In this space, lived experiences are co-constituted (they are not pre-given but arise from the interplay of bodies and attention, through synchrony and desynchrony). This concept emphasizes the emergent and non-dual nature of subjectivity, where self and other arise together. It has been described as **intercorporeality** (Merleau-Ponty, 1964), **homecomerade** (Steinbock, 1995), or **co-embodiment**, and serves as the experiential ground for **participatory sense-making** (De Jaegher & Di Paolo, 2007) and **interpersonal generative passages**.

**Intersubjectivity** (Zahavi, 2001; De Jaegher & Di Paolo 2007): Intersubjectivity refers to the capacity to share, co-create, and mutually shape lived experience through social interaction. It involves mutual coordination of meaning and experience over time and occurs *between individuals* rather than solely within them. The term is widely used in cognitive sciences to study how individuals connect and co-experience, often in development, therapy, or communication.

In contrast to **Interbeing Space**, which emphasizes the emergent, non-dual nature of subjectivity within a shared relational field, intersubjectivity highlights the interaction between distinct subjects and their coordination through embodied engagement.

**Intersubjective first-person perspective (1PP)**: When referring to intersubjective first-person perspective, we mean first-person experiential accounts that pertain to shared or relational dynamics between individuals. Phenomenological data are, by default, individually reported, which raises a methodological limitation that needs to be acknowledged when studying intersubjective or shared experience: we cannot directly access a “plural” or joint subjective report. However, this does not rule out the investigation of intersubjective dynamics or generative passages between embodied agents. A promising strategy builds on:

- Collecting first-person perspective from multiple participants included in the interaction or observing it and analyzing their resonances and divergences;
- Complementing subjective reports with relational self-report measures (e.g., *Inclusion of Other in the Self scale*) to evaluate the *experienced quality of the interaction;*
- Recognizing that an enactive and relational framework allows for sense-making to emerge from the interaction itself, even when only one participant reports it explicitly.

Such third-person approaches can then be used in tandem with intersubjective third-person perspective to operationalize generative passages between embodied agents.

**Intersubjective third-person perspective (3PP)**: When referring to intersubjective third-person perspective, we mean biobehavioral indicators of relational dynamics between individuals, as captured through external observation, measurement, or modeling. Unlike first-person perspective, which rely on self-report, third-person perspective includes physiological, behavioral, or neural signals. Common strategies to capture intersubjective third-person perspective include:

- **Simultaneous recording** of biobehavioral signals across several individuals in interaction, such as through hyperscanning or synchronized physiological monitoring;
- **Multimodal data acquisition**, integrating altogether measures like neural activity, heart rate, skin conductivity, respiratory rhythms, cortisol rates, and movement to assess coordination across many brain and body modalities;
- **Computational and statistical tools**, disentangling the complexity by revealing patterns of synchrony, coupling, and alignment over time or structure, particularly those robust to non-normal data distributions.

Such third-person approaches can then be used in tandem with intersubjective first-person perspective to operationalize generative passages between embodied agents.

**Lived Experience**: A term anchored in the enactive approach, which refers to what other types of neuroscientific studies of consciousness call **phenomenal consciousness** or **conscious experience**. It denotes the **circular dynamics between mind and body**. When studied in neurophenomenology, the term “emergence” points to the bidirectionality and circularity between mind and body. In this article, we propose a framework to explore how lived experience is impacted by and impacts social interaction with others.

**Mathematical Formalisms**: Abstract quantitative frameworks that provide a shared formal language for simulating, predicting, and comparing models across nested levels of organisation. For research on **body–brain–mind** processes and modeling generative passages at both individual and interpersonal levels, scale-agnostic formalisms are especially pertinent, including:

- **Dynamical Systems Theory**, characterises the temporal evolution of coupled variables through deterministic or stochastic flows, revealing attractors, bifurcations, and phase transitions.
- **Control Theory**, models how biological or artificial systems regulate their states through feedback, continuously comparing expected and actual outputs to minimise explicit cost functions. It formalises stability, error correction, and adaptive policy design in contexts ranging from cellular homeostasis to motor coordination and social regulation.
- **Bayesian Theory**, treats perception, cognition, and action as probabilistic inference, whereby prior beliefs are updated with sensory evidence to maintain and refine internal models from neural circuits to social interaction.
- **Information Theory**, quantifies the encoding, transmission, and degradation of signals within and between neural, behavioural, and social channels.
- **Network (Graph) Theory**, represents neural, bodily, or social elements as nodes and their interactions as edges, enabling the study of community structure, hubs, and information flow within and across scales.
- **Complexity Theory**, links microscopic interactions to macroscopic behaviour by describing large ensembles probabilistically, revealing emergent patterns in neural populations, physiological networks, and multi-agent collectives.

**Neurophenomenology (NPh)** (Laughlin et al., 1992; Varela, 1996): Neurophenomenology is a methodological framework introduced by Francisco Varela that integrates first-person perspective (1PP) experiential data with third-person perspective (3PP) neuroscientific observations, treating consciousness as a domain that demands both phenomenological rigor and scientific tractability (Varela, 1996). The term was originally coined in a different context by Laughlin, McManus, and d’Aquili in *Brain, Symbol & Experience: Toward a Neurophenomenology of Human Consciousness* (1992), and later reappropriated and developed by Varela to designate a research program grounded in enactive cognitive science and phenomenology.

**References**

De Jaegher, H., & Di Paolo, E. (2007). Participatory sense-making: An enactive approach to social cognition. *Phenomenology and the Cognitive Sciences, 6*(4), 485–507. <https://doi.org/10.1007/s11097-007-9076-9>

De Jaegher, H., Pieper, B., Clénin, D., & Fuchs, T. (2017). Grasping intersubjectivity: An invitation to embody social interaction research. *Phenomenology and the Cognitive Sciences, 16*(3), 491–523. https://doi.org/10.1007/s11097-016-9469-8

Dumas, G. (2011). *Neural dynamics of synchronous imitative interaction* (Doctoral dissertation, Sorbonne Université).<https://hal.science/tel-01123458>

Fodor, J. A. (1975). *The language of thought*. Harvard University Press.

Gallagher, S. (2003). Phenomenology and experimental design: Toward a phenomenologically enlightened experimental science. *Journal of Consciousness Studies, 10*(9–10), 85–99.

Husserl, E. (1966). *Analysen zur passiven Synthese* (M. Fleischer, Ed.). Husserliana XI. The Hague: Nijhoff.

Husserl, E. (1973). *Zur Phänomenologie der Intersubjektivität: Zweiter Teil* (I. Kern, Ed.). Husserliana XIV. The Hague: Nijhoff.

Laughlin, C. D., McManus, J., & d'Aquili, E. G. (1992). *Brain, symbol & experience: Toward a neurophenomenology of human consciousness*. Columbia University Press. (Original work published 1990)

Lutz, A., Abdoun, O., Dor‑Ziderman, Y., Trautwein, F. M., & Berkovich‑Ohana, A. (2025). An overview of neurophenomenological approaches to meditation and their relevance to clinical research. *Biological Psychiatry: Cognitive Neuroscience and Neuroimaging, 10*(4), 411–424. https://doi.org/10.1016/j.bpsc.2024.11.008

Marr, D. (1982). *Vision: A computational investigation into the human representation and processing of visual information*. W. H. Freeman.

Maturana, H. R., & Varela, F. J. (1992). *The tree of knowledge: The biological roots of human understanding* (Revised ed.). Shambhala.

Merleau-Ponty, M. (1964b). *The philosopher and his shadow* (R. C. McCleary, Trans.). In *Signs* (pp. 159–181). Evanston, IL: Northwestern University Press. (Original work published 1960)

Ramstead, M. J. D., Constant, A., Badcock, P. B., Friston, K. J., & Kirchhoff, M. D. (2022). Answering Schrödinger’s question: A free-energy formulation. *Physics of Life Reviews, 41*, 49–78. https://doi.org/10.1016/j.plrev.2022.01.001

Varela, F. J. (1996). Neurophenomenology: A methodological remedy for the hard problem. *Journal of Consciousness Studies, 3*(4), 330–349.

Varela, F. J. (1997). The naturalization of phenomenology as the transcendence of nature: Searching for generative mutual constraints. *Alter, Revue de Phénoménologie*, (5), in press.

Varela, F. J. (1999b). Steps to a science of interbeing : Unfolding implicit the dharma in modern cognitive science. In The psychology of awakening : Buddhism, science and our day to day lives (Bachelor S, Claxton G, Watson G, p. 71‐89). Rider/Randol House.

Zahavi, D. (2001). Beyond empathy: Phenomenological approaches to intersubjectivity. *Journal of Consciousness Studies, 8*(5–7), 151–167.
